# Understanding the Impact of the Psychological Cognitive Process on Student Learning Satisfaction: Combination of the Social Cognitive Career Theory and SOR Model

**DOI:** 10.3389/fpsyg.2021.712323

**Published:** 2021-08-26

**Authors:** Guihua Zhang, Xiaoyao Yue, Yan Ye, Michael Yao-Ping Peng

**Affiliations:** ^1^Department of Business, Yeungnam University, Gyeongsan, South Korea; ^2^Graduate School of Human Sciences, Assumption Universtiy, Bangkok, Thailand; ^3^Graduate School of Education, Stamford International University, Bangkok, Thailand; ^4^School of Economics & Management, Foshan University, Foshan, China

**Keywords:** generic skills, interaction relationship, learning satisfaction, PLS-SEM, social support, self-efficacy

## Abstract

In higher education, student learning satisfaction is a significant predictor of learning that indicates the commitment students have to their learning and future academic achievement. The study combines the social cognitive career theory (SCCT) and the stimulus-organism-response (SOR) model to explore the psychological cognition and attitudes derived from students during their learning, discusses the pattern of student learning satisfaction enhancement from the aspect of process, and further understands the relationships among social support systems, interaction relationships, self-efficacy, generic skills, and learning satisfaction. In this study, 800 valid copies of questionnaires were collected from 12 universities through purposive sampling, and the structural model was analyzed by partial least squares structural equation modeling (PLS-SEM). The results showed that the relationships among all the constructs were positive and showed a significant effect; furthermore, the research results showed that self-efficacy and student generic skills had a significantly indirect effect in the model—specifically, a mediating effect. Finally, corresponding theoretical and practical implications were put forward based on the research results.

## Introduction

Student learning has always been valued by scholars, especially in discussing how to enhance student learning effectiveness and learning engagement (Pike et al., 2011, 2012; Peng and Chen, 2019; Li et al., 2020; Peng et al., 2021). Past studies have stated that better learning effectiveness represents students with strong learning motivation and commitment, which are reflected in their learning achievements because of their learning preferences (Pike et al., 2011; Li et al., 2020). Self-determination theory mentions that students can decide their own roles in learning and have a high degree of intrinsic motivation and autonomy to understand the importance of learning and improve learning effectiveness (Vallerand et al., 1997; Shogren et al., 2014; Sergis et al., 2018). However, although Western theories emphasizing intrinsic motivational factors have proven their importance in Eastern society, the cultural differences in Asia make students more likely to face the social expectations of their families and other interpersonal relationships, thus forcing themselves to learn in conformity with the expectations of family members (Chang et al., 2011; Marambe et al., 2012; Li et al., 2020). Although most students pursue differences in grades and performance, it is more important for them to find their own preferences and interests in learning and cultivate their professional capabilities and knowledge base; therefore, learning satisfaction is another psychological dimension of learning effectiveness. Furthermore, learning satisfaction also reflects the effects of the learning students engage in. Liu et al. (2020) used social cognitive career theory to discuss the employability of students and replace the discussion of learning effectiveness in these students with actual skill growth (Peng, 2019). Different from past research, learning satisfaction can be used to determine the psychological state of the learning that students have (Kong and Yan, 2014; Pan, 2014) and construct a vital source of future learning motivation (Oyarzun et al., 2018; Alqurashi, 2019); in other words, the higher the learning satisfaction, the higher the intrinsic motivation and actual learning effectiveness (Yilmaz, 2017). Therefore, this research will explore the pre-variables of learning satisfaction and understand how to promote student learning satisfaction.

In regard to the study of learning satisfaction, since Lent and Brown proposed the Social Cognitive Career Theory (SCCT) in 2006, many scholars began to build a research framework based on the SCCT model for exclusive research situations (Lent et al., 2017; Lent and Brown, 2019; Liu et al., 2020; Lee et al., 2021; Pandita et al., 2021). Peng et al. (2021) used the SCCT model to conduct a cross-cultural comparative analysis, using teacher knowledge transfer as a pre-variable to explore the relationship among model variables. Although the SCCT model is widely used by scholars to explore the cognitive influence path of individuals facing external environmental stimuli, it rarely mentions the evolution process of the mental state (Lent and Brown, 2013; Park et al., 2018; Zhai et al., 2020). Mehrabian and Russel (1974) proposed the SOR model, which pointed out that all individuals' behavioral responses or psychological changes are stimulated by the external environment, and the individual will inductively process the stimulus and adjust the psychological interaction to produce an appropriate response (Zhai et al., 2020; Pandita et al., 2021). The SOR model describes the connection between stimuli (such as external factors) that will affect organisms (cognition and emotion of people) and the response people have to the stimulus (such as behavior). Stimulus (S) refers to input, which is an external factor related to the environment. Organisms are things that will respond to stimuli (Eroglu et al., 2003), which include emotions, feelings, and emotions to these stimuli. Reaction (R) refers to actions and reactions students have to organisms (Buxbaum, 2016). Human beings are organisms that produce emotional and psychological elements and the mood, emotions, or attitudes that respond to stimuli; thus, the stimulus-organism-response (SOR) model has been extended (Zhai et al., 2020). In the context of this research, social support systems and interaction relationships are conceptualized as stimuli, self-efficacy and generic skills are the dominant organisms, and student satisfaction is the response. The process of student participation stems from the stimulation of the learning environment (Hazeltine and Schumacher, 2016). Therefore, this study will build an SOR model based on the variables of the SCCT model and explore student satisfaction by combining the characteristics of the two models. Scholars believe that the setting of pre-variables will affect the subsequent psychological response of the individuals (Zhai et al., 2020), while most of the stimulus variables studied in the past emphasize the external and internal influences that affect the learning of students in the classroom; thus, the research context focused on classroom level (Yang et al., 2021). However, whether or not the psychological cognitive results will remain the same or similar after students leave the classroom, there is an unsolved black box (Wong, in press). In order to avoid the impact of endogenous variation that may be brought about by the pre-variables designed at the classroom level, this research will propose important external pre-factors from the school level (Ghosh and Fouad, 2017; Zhang et al., 2018) to further enhance the generalization of the research, including the campus social support system and student interaction.

The school is a small social system, and student life, the process of learning, and peer communication in the school continuously affect the quality of student learning and engagement degree (Mattanah et al., 2012). Scholars believe that the higher the input in learning support, the greater the motivation and intention of students to engage and the improve how they adapt to campus life (Matsuda et al., 2014; Ghosh and Fouad, 2017). Similarly, scholars pointed out that most of the situations in which students feel powerless or helpless in learning may come from their inability to feel the care they have for learning, and the inability to obtain effective support for difficult-to-understand courses (Hen and Goroshit, 2014; Yssel et al., 2016). Thus, if the peer learning interaction is low, it may cause a vicious circle of the Matthew effect (Otto and Kistner, 2017). Therefore, the social support system in school and interaction relationships can be used to explore the important external pre-factors affecting student learning satisfaction and the stimulating variable roles of the two in the SOR model.

**Figure 1:**
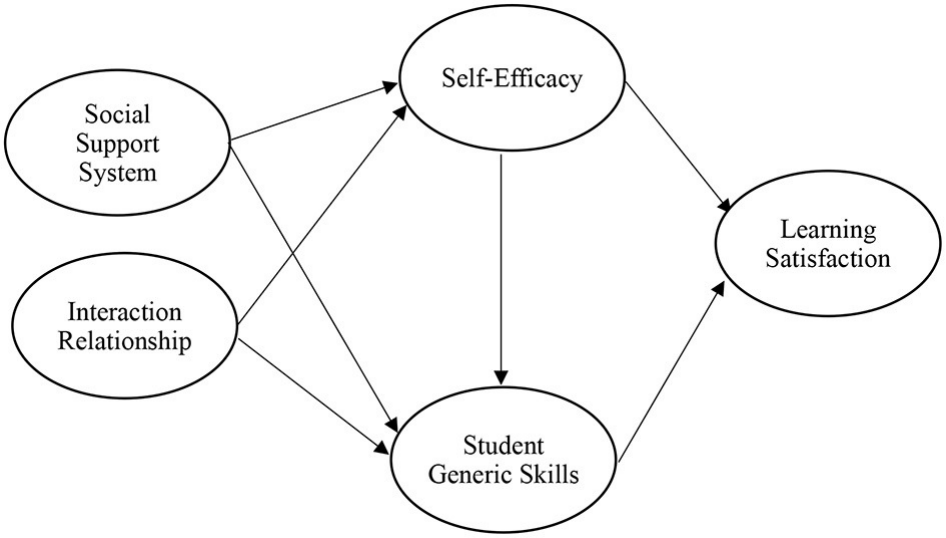
Research framework.

External support systems and interaction relationships may have a significant impact on learning satisfaction, but whether in the SOR or the SCCT model, these systems still need to undergo transformations in their internal mechanisms or psychological cognitive factors (Isik, 2013; Chan, 2020) to form a clear relationship. In the SCCT model, self-efficacy is a key cognitive factor that acts as an intermediary bridge between environmental factors and satisfaction (Hen and Goroshit, 2014; Chan, 2020); as a satisfaction model constructed through self-efficacy, it can also strengthen the overall effect of the preceding factors on the dependent variables. In other words, individuals with a high degree of self-efficacy can effectively identify the resources of the external environment and leverage them to solve or perform real-world problems and tasks (Liu et al., 2020; Lee et al., 2021). In addition to self-efficacy, students also need to recognize the knowledge, skills, and basic literacy they have learned (Coates and Richardson, 2012; Tremblay et al., 2012), which reflects the substantial effects of the pre-factors; especially in the SCCT model, cognitive learning output is an important intermediary variable that highlights the influence of pre-variables and self-efficacy on learning satisfaction. Therefore, this study will further explore the mediating effects of the self-efficacy and generic skills of students in the model. Based on above arguments, this study provides a conceptual framework as Figure 1.

## Literature Review

### SCCT and SOR Model

The social cognitive career theory model is based on the social cognitive theory (SCT) of A. Bandura by Lent et al. (1994). It is divided into three models, and they are the “interest model” that is fond of a certain career field, the “selection” model that converts interest into specific career intentions, and the “career achievement model” that chooses to enter a certain career field to show professional performance (Lent and Brown, 2013, 2019). Therefore, the SCCT considers the interaction between the individual, environment, and behavior to explain the formation of professional interests, planning of personal educational choices and career directions, and choice of a certain professional field of achievement performance, etc. (Lent et al., 2008; Liu et al., 2020; Lee et al., 2021). Since the subject of this research is college students, it focuses on the interest model and the selection model. However, the SCCT model seldom mentions the changes in the psychological cognition of students during the learning process, especially the external factors that affect psychological cognition. The SCCT model emphasizes the interaction of context on preferences and choices. When the SCCT was introduced 25 years ago, the theory initially included (a) career and academic interest development, (b) choice, and (c) performance. It was later expanded to include two additional models, with one focusing on education and career satisfaction or happiness and the other focusing on the process of self-management throughout the career life cycle (Brown and Lent, 2019). The SCCT explained that the intention to pursue a specific goal in a career comes from the judgment a person has on what they think is feasible (self-efficacy) and the possible impact of their expected actions (outcome expectations) (Bandura, 1989). In addition, the concept of outcome expectations can be further subdivided into internal and external aspects (Lanero et al., 2016). Internal result expectations refer to factors related to personal accomplishment, work, independence, and learning opportunities. On the other hand, external result expectations refer to economic remuneration, job security, and social recognition.

The SOR model consists of three structures—namely, stimulus, organism, and response—, which determine the behavioral outcome of an event. The concept of stimulus and response is described as “a part of behavior and environment.” Sudden changes in the environment will affect the psychological and emotional stability of an individual, thereby further promoting changes in their behavior. Stimulus is defined as “influencing the individual,” and is the external force that affects the mental state of an individual (Fu et al., 2021). An organism can be referred to as the internal process and structure between the external stimulus of a person and their final action, reaction, or response. The intervention process and structure include perceptual, physiological, sensory, and thinking activities (Pandita et al., 2021). In the field of environmental psychology, the SOR model explains that various external factors can be used as stimuli (S), which in turn affect the internal state of the individual (O), and thus the behavioral response exhibited by the individual® (Zhai et al., 2020; Fu et al., 2021). On the basis of the SCCT model, adding the concept of the SOR model will help this study explain the changes in the mental cognition of students during the learning process and their subsequent learning intentions and behavioral responses. The SOR model helps explain the internal psychological changes caused by the individual being stimulated by the environment (Lin et al., 2020).

In the context of this research, the social support system and interaction are conceptualized as stimuli, self-efficacy and basic literacy are the dominant organisms, and student satisfaction is the response. The process of student participation stems from the stimulation of the learning environment (Hazeltine and Schumacher, 2016). Since the subject of this research is college students, it focuses on the interest model and the selection model. However, the SCCT model seldom mentions the changes in the psychological cognition of students during the learning process, especially the external factors that affect psychological cognition. The SCCT model emphasizes the interaction of context on preferences and choices.

### Learning Satisfaction

Satisfaction is the perception of the difference between previous expectations and perceived achievement (Nagy, 2018). Keller (1983) defines learning satisfaction as the overall positive evaluation of a student of his or her learning experience (Bunce et al., 2017; Li et al., 2017; Hew et al., 2020). Satisfaction can only be measured after learning activities (Li, 2018; Nagy, 2018). Li (2018) pointed out that learning satisfaction is the feeling and attitude toward the learning process; this feeling and attitude are formed by the joy that students feel when their learning activities or learning process meet their physical and psychological needs. Nelson (2016) regards learning satisfaction as a combination of good perception and positive attitude. This is because learning activities can meet personal needs; that is, learners can perceive the satisfaction of personal learning needs during the learning process. Emtinan (2018) pointed out that student satisfaction reflects how learners perceive their learning experience (Keller, 1983; Li et al., 2017; Weidlich and Bastiaens, 2017). The importance of the learning satisfaction of students is highly correlated with the dropout rate, determination, motivation, and determination of these students to complete a degree and succeed.

The self-efficacy of college students is significantly related to student satisfaction. Learner satisfaction reflects the perceptions students have of their learning experience (Emtinan, 2018). Satisfaction is the basic result of learners because it can affect their motivation level, which, in turn, is an important psychological factor that affects the learning of students. Learner satisfaction is an important dependent variable because it has a strong positive correlation with the perceived teaching quality of learners, especially in the traditional university learning environment (Hew et al., 2020). The suggestion of learning satisfaction as an important outcome is also consistent with recent marketization forces, which treat students as consumers of educational products or services (Bunce et al., 2017).

### Self-Efficacy

The SCCT has accumulated numerous empirical studies, showing that the self-efficacy of individual variables, the expectation of results, and the interest in learning can strengthen the investment a person has in a certain field, with self-efficacy being the most critical variable (Lent et al., 1994, 2010; Liu et al., 2020; Lee et al., 2021). The individual effectiveness not only affects how they think, feel, motivate, and then act, but it is the process that also affects how individuals choose behaviors, how much effort they are willing to put into execution, and how much emotion and pressure they can bear (Pan, 2014; Chan, 2020). Self-efficacy refers to the ability of an individual to judge how to complete a specific task or action, and it is also one of the most important self-regulatory mechanisms that affect individual behavior. In other words, self-efficacy means a subjective judgment of the ability of an individual to organize a plan before actual action in order to achieve a certain goal (Hen and Goroshit, 2014; Pan, 2014). When individuals have high self-efficacy, they are willing to set higher goals when faced with tasks, are less afraid of failure, and will persist to overcome obstacles when encountering difficulties; on the contrary, when the self-efficacy of these individuals are low, they will be reluctant to really take action, and when faced with difficulties, they will easily give up and not want to continue to persevere (Erdem and Demirel, 2007).

Previous studies have provided strong evidence that self-efficacy is a positive predictor of performance outcomes for different subjects. Self-efficacy “can predict students' academic performance in various fields and levels (Lent et al., 2008; Liu et al., 2020; Lee et al., 2021).” There is a large body of evidence to support the direct impact of self-efficacy beliefs on academic performance (Doménech-Betoret et al., 2017). Lee and Mendlinger (2011) indicated that perceived self-efficacy serves as an antecedent to learning satisfaction and has a positive effect. Good academic performance improves the self-confidence of students in learning, and, in turn, their self-efficacy. Therefore, self-efficacy is a powerful predictor of learner satisfaction. Based on the above discussion, the following hypothesis can be obtained:

*H1: The self-efficacy of students is positively correlated to their learning satisfaction*.

### Student Generic Skills

Generic skills can be regarded as generic attributes, key skills, and core competencies. They are widely mentioned in the community, education, and work-life. In addition to discussing from the perspective of students, they also include human resources. Generic skills have also been included in national and international qualification frameworks such as the European Qualifications Framework (EQF), clearly indicating any knowledge, skills, and competencies recognized by the learner (European Parliament and European Council, 2008). Many scholars also emphasize that generic skills can be used to compare the education situation between countries and provide directions for improving the quality of teaching and learning (Coates and Richardson, 2012; Tremblay et al., 2012). Studies have even pointed out that generic skills can be used as key skills that students need to have in the labor market in the future. Even in different majors and disciplines, they must have such general skills, such as organizing skills, knowledge acquisition, and problem-solving skills (Tynjälä et al., 2006; Virtanen et al., 2009; Arevalo et al., 2010). Although generic skills are not as important as employability and other abilities for task execution in the workplace, this ability reflects the intuitive response the learner has to daily life, as well as their views and insights on problems. Therefore, in this study, the concept of generic skills will be used as an important skill for students to improve upon through external stimuli during the learning process. Virtanen and Tynjälä (2018) pointed out that the essence of studying generic skills is that it can improve the existing curriculum design and learning environment and enable students to have a deeper understanding of their self-concept and self-role. Students with higher self-efficacy tend to be more engaged, work harder, spend a substantial amount of time trying their best to complete duties (Chan, 2020), pursue challenging goals, and become hardworking. Researchers believe that self-efficacy may affect learning motivation and increase academic achievement (Hsieh et al., 2007). The more sense of self-efficacy students have, the more willing they will be to spend their energy on learning; thus, they can master more generic skills. Satoshi et al. (2009) shed light on the self-efficacy of generic skills students have as a new measure for learning outcomes. The study also provided empirical evidence of possible correlations between the self-efficacy of generic skills students have and their choice of a major. In addition to developing abilities and acquiring the skills to perform course tasks, students need to establish a strong belief that they can successfully complete these tasks (Chan, 2020). Therefore, it seems that the self-efficacy component of motivation reflects positive academic performance (Komarraju et al., 2010). Based on the above discussion, the following hypothesis can be obtained:

*H2: The self-efficacy of students is positively correlated to their generic skills*.

Generic skills have hidden characteristics, which are different from subject-specific knowledge or hard skills. These skills emphasize the cognitive and emotional growth of students (Zepke and Leach, 2010; Freudenberg et al., 2011). In the dynamic teaching process, teachers guide students into interactive social processes (Jones, 2009; Virtanen and Tynjälä, 2018), by creating social contexts to support the learning process of students and maintain relationships with them (Barrie et al., 2009). Students continuously convert and extend conceptual skills and knowledge in the classroom tasks set by the teacher, and thus obtain substantive generic skills through close collaboration and social interaction with their classmates (Precision Consultancy, 2007). When students recognize that generic skills have been substantially improved, it means that there is a pleasant learning atmosphere in the classroom, which not only improves the knowledge of the exclusive subject but also improves the positive view a student has of their self-concept (Freudenberg et al., 2011). This is further reflected in learning satisfaction. Teo et al. (2012) noted that students who have received training in group work, such as generic skills, are more likely to report a high level of satisfaction with the peer evaluation process in the group work assessment task. Therefore, the hypothesis of this research is as follows:

*H3: The generic skills of students are positively correlated to their learning satisfaction*.

### Social Support System

Since the mid-1970s, there has been an increasing interest in social support as a coping factor related to physical health (Bruwer et al., 2008; Ermis-Demirtas et al., 2018). Social support has been regarded as a multidimensional construct and defined in various ways (Cobb, 1976; Kang and Nancy, 1996; Williams et al., 2004; Bruwer et al., 2008; Ellonen et al., 2008; Vollmann et al., 2010). Social support is defined as the perception a person has of specific or general supports from people in their context, which contribute and/or act as a buffer for their wellbeing (Demaray and Malecki, 2002; Malecki and Demaray, 2003; Vedder et al., 2005; Marambe et al., 2012; Ermis-Demirtas et al., 2018; Wilson et al., 2020). Especially in adolescents research, social support is regarded as a manifestation of the community (Ellonen et al., 2008; Lippman et al., 2014). Perceived social support can also be related to wellbeing (Rosenfeld et al., 2000; Vedder et al., 2005; Haber et al., 2007; Camara et al., 2017; Fogaca, 2021). Furthermore, poor social support could predict low levels of outcomes in the psychology and academics of students (Rosenfeld et al., 2000; Malecki and Demaray, 2003; Haber et al., 2007). Social support is considered a social resource, social asset, or social network that people can use when they need help, assistance, advice, approval, protection, comfort, or support. It covers information that a person cares about, respects, and values, is part of a network of communication, and is a two-way responsibility (Cobb, 1976).

Vollmann et al. (2010) found social support to be the most beneficial in reinforcing student self-esteem (Camara et al., 2017). According to Kang and Nancy (1996), students, as customers of the universities, have a need for social support. Social support is an important dimension in improving self-efficacy (Maleki-Saghooni et al., 2020). The self-efficacy of a person is positively correlated with the social support they receive. In other words, the more social support a person receives, the higher their sense of self-efficacy (Wang et al., 2015). Social support plays an important role in the vigorous development of the entire life cycle, especially during periods of change, such as the dramatic changes that represent adolescence (Ellonen et al., 2008; Lippman et al., 2014). Past research on adolescents has shown that perceived social support is significantly correlated with positive emotions and high activeness. On the contrary, perceived social support is negatively related to the internalization and externalization of negative emotions and adolescent symptoms, including aggressiveness. Social support can increase the self-esteem and self-confidence of adolescents (Orkibi et al., 2018). Liu et al. (2020) and Xu et al. (2021) also showed that social support from teachers and peers has significantly positive correlations with self-efficacy. Based on the above reasons, the following hypothesis is made:

*H4: The social support systems of students are positively correlated to their self-efficacy*.

Researchers have discovered the relationship between perceived social support and various academic achievements. There is an association between social support and academic indicators (for example, grades, standardized achievement tests, and teacher ratings). The relationship between social support and academic performance of adolescents (such as attendance, avoidance of problem behaviors, grade level, prosocial behaviors, school satisfaction, and school continuity) positively facilitates learning engagement. In academic research, a relationship was found between various specific supports (for example, listening and emotional support) and positive learning outcomes (Malecki and Demaray, 2003). A large body of research shows that there is a positive correlation between social support and results that educators are particularly interested in, such as student motivation, school adaptation, school belonging, dropout rate, ability to deal with daily school troubles, especially learning and academic behavior. The more social support a student receives, the higher level of generic skills of the student. In addition, social support directly or indirectly improves the academic performance and abilities of students, including test scores and usual results (Rosenfeld et al., 2000). As a result, the following hypothesis is formed:

*H5: The social support systems of students are positively correlated to their generic skills*.

### Interaction Relationship

The interaction relationship is intended to establish a good tacit understanding and consensus among learners in the process of contact, exchange, and communication with others in the learning environment (Pike et al., 2012; Kim and Lundberg, 2016; Peng, 2019). In social capital, interpersonal interactions play an important role of contact (Carton and Goodboy, 2015; Brouwer et al., 2016; Peng, 2019). Through interaction relationships, individuals can strengthen their sources of information and knowledge in social networks, consolidate the links between existing relationships, and make information transmission in social networks smoother (Komarraju et al., 2010). Any individual contact and communication encountered by students were playing an important role in the learning process, such as teachers, classmates, administrative staff, etc. (Komarraju et al., 2010; Kuo et al., 2014; Kim and Lundberg, 2016). Komarraju et al. (2010) pointed out that students with good interaction relationships can more easily adapt to campus life and acquire more information and knowledge needed for learning (Han et al., 2020), which can strengthen positive mental cognition and substantive skills acquisition of these students (Martin and Rimm-Kaufman, 2015). Kim and Lundberg (2016) pointed out that the interaction relationship between students and teachers will encourage students to derive higher academic engagement; thus, having the motivation to challenge themselves and then produce and acquire good learning results and skills (Bowman and Park, 2015).

Scholars pointed out that the establishment and maintenance of social relationships help individuals (students) to integrate into various groups and obtain valuable information and knowledge in each of their social networks (Martin and Rimm-Kaufman, 2015; Brouwer et al., 2016; Han et al., 2020). All relationships must be established through interaction. If students have strong interaction relationships, they can perceive any available resources to complete their course tasks and face learning challenges more confidently in the learning process (Kuo et al., 2014; Martin and Rimm-Kaufman, 2015). Related research has pointed out that the stronger the social and interaction relationship of students, the stronger their self-efficacy in learning skills and knowledge acquisition (Wang et al., 2015; Brouwer et al., 2016). Xu et al. (2021) indicated that students with more social capital from peers/teachers are likely to be more involved in their learning environment and actively participate in learning activities, thus improving self-efficacy. Based on the above description, the inference assumption is as follows:

*H6: The interaction relationships of students are positively correlated to their self-efficacy*.

In many studies, it has been pointed out that the interaction relationship between students and teachers has a significant positive correlation with learning effectiveness in students. Tynjälä et al. (2016) studied the social competence of students in a Finnish university. Based on the socio-cultural approach, they used Interaction Skills in a Group and in Networks (ISGN) and Social and Emotional Skills in Teaching (SEST) to establish students with good social relations. In the learning community, students shared knowledge with each other and developed collaboration to complete tasks (Han et al., 2020). With the intervention of social cognitive psychology and philosophical diagnosis, students were guided to strengthen their interaction with each other, so as to enhance their initiative to participate and gain more substantial experience and intuitive responses to problems (Zepke and Leach, 2010). Therefore, students with stronger interaction relationships can change their personality traits according to the social environment of different tasks. Under the change of adjustment ability, the generic skills of students will also improve (Pike et al., 2011). Therefore, based on the above content, the inference assumption is as follows,

*H7: The interaction relationships of students are positively correlated to their generic skills*.

Based on hypotheses 1–7, we developed the research question as follows: What is the relationship between learning satisfaction and self-efficacy, generic skills of students, social support systems, and interaction relationships based on the SCCT model and SOR model?

## Methodology

### Sampling

The purposes of this research are to explore the learning satisfaction of students in the learning process and analyze the impact of the social support provided by the school and the interaction relationship on students. The research sample in this study comprised undergraduates. Purposive sampling was adopted. However, this sampling suffers from several disadvantages. Vulnerability to errors in judgment by researchers, low level of reliability and high level of bias, and inability to generalize research findings are the three main disadvantages of purposive sampling. To avoid these disadvantages, some conditions were set during sampling in this study to make the samples obtained better conform to sample reliability and, therefore, improve the generalization of the study. Since the sampling objects were college students and the number of maternal populations was huge, in order to make the research results closer to the issues that this research study intended to explore, some sampling conditions were set during the sampling process. First of all, as subject differences may have an impact on student learning, in order to reduce the impact of the subject on this research model, the subjects were divided into two categories: social sciences and natural sciences. The samples of the two subjects were collected on average. Second, since the cognition of the interaction relationship and the social support system takes time to be felt, the sample did not include freshmen; only sophomores, juniors, and seniors were collected. This study selected 12 Taiwanese universities and then sent 2,000 questionnaires to them. After sampling, a total of 800 questionnaires were returned for an effective response rate of 40%. Since freshmen were not familiar with the learning environment, all participants in this study were sophomore, junior, and senior students. Table 1 shows the descriptive statistics of the samples.

**Table 1:** Descriptive statistics.

**Characteristic**	**Scale**	**n**	**Percentage**
Gender	Male	453	56.6
	Female	347	43.4
Part-time job	Yes	488	61.0
	No	312	39.0
Scholarship	Yes	322	40.2
	No	478	59.8
First-generation college student	Yes	433	54.1
	No	367	45.9
Majors	Social science	423	52.9
	Natural science	377	41.1.
Dedication to class preparation	Yes	336	42.0
	No	464	58.0

Due to the different genders and types of disciplines, a systematic error might have arisen, bringing the external validity of the study into question. Thus, several independent-samples *t*-tests were used to verify whether the groups of male vs. female and social sciences vs. natural sciences differed significantly in terms of research dimensions. The results indicated that the groups did not significantly differ, so it was deemed appropriate to merge the samples from different genders and disciplines.

### Measures

Most of the scales in the questionnaire were adopted from previous studies and modified to suit the research context. In studying the social support system, four items were developed on the basis of a prior scale and item analyses with Asian applications (Ryan, 2004). To divide interaction relationships into student-faculty interaction (four items) and interpersonal environment (three items), we adopted the scale proposed by Pike et al. (2012). The scale is based on the characteristics of undergraduates in Western countries, such as the US, and its credibility and validity have been verified; therefore, we found the scale suitable for expansion to the Asian context. Self-efficacy can be referred to as the degree of the perceptual ability of an individual to achieve a goal. The scale was revised to integrate six items of higher reliability and validity by Rigotti et al. (2008). For generic skills, students were asked to evaluate themselves with an instrument proposed by Freudenberg et al. (2011). The instrument adopted 10 broad skills, nine of which describe commonly identified areas of generic skills, such as interpersonal skills, self-management skills, learning and adaptability skills, problem-solving skills, concept and analysis skills, oral communication, team skills, information literacy skills, and written communication skills.

Learning satisfaction measurement items were adopted based on a previous scale (Hong et al., 2016) and focused on the satisfaction degree of undergraduate students with their learning process and environment, including 5 items. All items were measured with a five-point Likert scale (1 = totally disagree; 5 = totally agree) and are shown in Table 2.

**Table 2:** Instruments description.

**Construct**	**Variables**	**Items**
Social support	Social support	I can feel the instructional resources input by the school
		I can feel the resources of academic support input by the school
		I can feel that the school has an explicit input of resources in serving students (the efficiency of the administrative department)
		I can feel the school's dedication to enhancing students' well-being
Interaction relationship	Student-faculty interaction	Discussed grades or assignments with an instructor
		Talked about career plans with a faculty member or advisor
		Discussed ideas from your readings or classes with faculty members outside of class
		Worked with faculty members on activities other than coursework
	Interpersonal environment	Developed a good relationship with other classmates
		Developed a good relationship with teachers
		Developed a good relationship with administrative staff and offices
Self-efficacy	Self-efficacy	I can remain calm when facing difficulties in my job because I can rely on my abilities
		When I am confronted with a problem in my learning tasks, I can usually find several solutions
		Whatever comes my way in my learning tasks, I can usually handle it
		My past experiences in my learning tasks have prepared well for my occupational future
		I meet the goals that I set for myself in my learning tasks
		I feel prepared for most of the demands in my learning tasks
Generic skills	Generic skills	Teacher makes me proud to being associated with him/her
		Teacher has a “sense of mission” which he/she transmits to me
		Teacher displays conviction in his/her ideas, beliefs, and values
		Teacher specifies the importance of having a strong sense of purpose
Learning satisfaction	Learning satisfaction	Course contents inspired me to learn more professional skills
		Course contents solved past problems I had when learning my major
		The interactive style of course contents improved my professional skills
		Course contents make me want to continue learning from it
		I enjoy course contents with peers while we improve our professional skills together

## Results

### Assessment of Measurement Model

All scales used in this study were found to be reliable, with Cronbach's α ranging from 0.83 to 0.96. Table 3 shows the reliability of each scale and the factor loadings for each item therein. In order to gauge validity, this study employed confirmatory factor analysis (CFA) using AMOS 23.0 to verify the construct validity (both convergent and discriminant) of the scales. According to Hair's et al. (2010) recommended validity criteria, CFA results show standardized factor loading of higher than 0.7; average variance extracted (AVE) ranges between 0.539 and 0.729; composite reliability (CR) ranges between 0.8 and 0.918. All three criteria for convergent validity were met, and correlation coefficients were all less than the square root of the AVE within one dimension, suggesting that each dimension in this study had good discriminant validity.

**Table 3:** Measurement properties.

	**1**	**2**	**3**	**4**	**5**	**6**	**7**	**8**
1 Social support								
2 Faculty	0.541							
3 Peer	0.596	0.625						
4 Self-efficacy	0.556	0.409	0.485					
5 Creativity	0.383	0.355	0.378	0.472				
6 Critical think	0.462	0.408	0.407	0.504	0.771			
7 Meta cognition	0.425	0.480	0.359	0.427	0.619	0.749		
8 Learning sati	0.503	0.520	0.481	0.528	0.414	0.458	0.431	
Mean	3.695	3.237	3.608	3.746	3.439	3.429	3.252	3.448
SD	0.635	0.816	0.708	0.625	0.764	0.730	0.794	0.774
α	0.926	0.925	0.815	0.898	0.869	0.869	0.818	0.900
AVE	0.604	0.816	0.730	0.662	0.884	0.719	0.846	0.716
CR	0.938	0.947	0.890	0.922	0.938	0.911	0.917	0.926

### Testing Structural Model Fit

Before proceeding to examine the structural model, we first tested the model fit. Henseler et al. (2015) proposed three model fitting parameters: the standardized root mean square residual (SRMR), the normed fit index (NFI), and the exact model fit. According to Henseler et al. (2015), the evaluation standards for convergent validity are (1) the NFI should be larger than 0.9, (2) the SRMR should be <0.08, and (3) the exact model fit, which tests the statistical (bootstrap-based) inference of the discrepancy between the empirical covariance matrix and the covariance matrix implied by the composite factor model. Dijkstra and Henseler (2015) suggested the *d_LS* (squared Euclidean distance) and *d_G* (geodesic distance) as two different ways to compute this discrepancy. Henseler et al. (2015) indicated that *d*_*ULS*_ and *d*_*G*_ < than the 95% bootstrapped quantile (HI 95% of *d*_*ULS*_ and HI 95% of *d*_*G*_).

In this study, the SRMR value was 0.063 (<0.08), the NFI was 0.912 (>0.90), and the *d*_*ULS*_ < bootstrapped HI 95% of *d*_*ULS*_ and *d*_*G*_ < bootstrapped HI 95% of *d*_*G*_, indicating the data fits the model well.

### Inner Model Analysis

Partial least squares structural equation modeling (PLS-SEM) was adopted to construct the structural model; specifically, the verification of the structural model was performed using SmartPLS 3.0 (path analysis). To assess the structural model, Hair et al. (2017) suggested looking at the *R*^2^, beta (β), and the corresponding *t*-values *via* a bootstrapping procedure with a resample of 5,000. They also suggested that, in addition to these basic measures, researchers should also report the predictive relevance (Q^2^) as well as the effect sizes (f^2^). Prior to hypotheses testing, the values of the variance inflation factor (VIF) were determined. The VIF values were <5, ranging from 1.377 to 2.274. Thus, there were no multicollinearity problems among the predictor latent variables (Hair et al., 2017).

**Figure 2:**
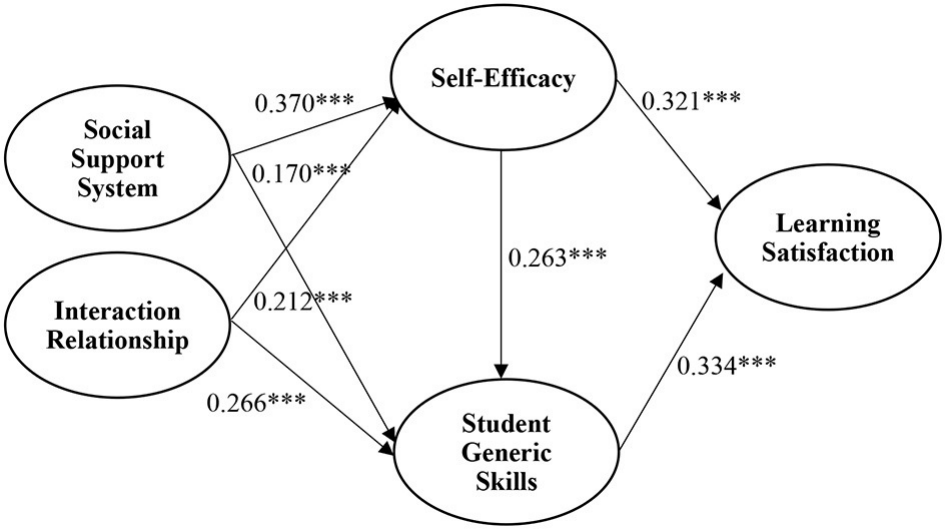
Structural model. ***p < 0.001.

Figure 2, Table 4 show the results of the hypothesized relationships and standardized coefficients in the inner model. The results showed that a social support system was positively and significantly related to student self-efficacy (β = 0.370, *p* < 0.001) and student generic skills (β = 0.170, *p* < 0.001), supporting H1 and H2. Similarly, interaction relationships were positively and significantly related to student self-efficacy (β = 0.212, *p* < 0.001) and student generic skills (β = 0.266, *p* < 0.001), supporting H3 and H4. In addition, our results found that student self-efficacy was positively and significantly related to student generic skills and learning satisfaction, supporting H5 and H6. Finally, student generic skills were positively and significantly related to student learning satisfaction, supporting H7. The Stone-Geisser Q2 values obtained through the blindfolding procedures for student self-efficacy (*Q*^2^ = 0.184), student generic skills (*Q*^2^ = 0.266), and student learning satisfaction (*Q*^2^ = 0.222) were larger than zero, supporting the predictive relevance of the model (Hair et al., 2017).

**Table 4:** Results of the hypotheses testing.

**Paths**	**Std. β**	**Std. error**	***t*** **-value**	**Decision**	**Significance CI (2.50–97.5%)**	**VIF**	***f*** ^**2**^
H1: SSS → SE	0.370	0.042	8.757	Support	CI (0.291–0.453)	2.274	0.114
H2: SSS → SGS	0.170	0.044	3.860	Support	CI (0.081–0.256)	2.452	0.023
H3: IR → SE	0.212	0.043	4.888	Support	CI (0.125–0.292)	2.274	0.037
H4: IR → SGS	0.266	0.042	6.389	Support	CI (0.182–0.347)	2.393	0.061
H5: SE → SGS	0.263	0.040	6.537	Support	CI (0.180–0.340)	1.512	0.074
H6: SE → LS	0.321	0.036	9.009	Support	CI (0.254–0.392)	1.377	0.117
H7: SGS → LS	0.334	0.035	9.563	Support	CI (0.265–0.401)	1.377	0.127

*CI, Confidence intervals (Lower bound—Upper bound)*.

### Examination of Mediating Effects

To establish a structural model, self-efficacy and student generic skills in the SCCT and SOR models can be regarded as intermediary variables. In order to understand whether the two have intermediary effects, a bootstrapping procedure was further carried out on the structural model. Results displayed in Table 5 indicate that the indirect effects of self-efficacy and student generic skills were supported. It shows that the setting of important intermediary variables plays an important role in either the SCCT model or the SOR model. In particular, self-efficacy, similar to the results of previous studies, can highlight the effects of pre-variables in the model, forming strong intrinsic motivation and cognition, which are then reflected in the outcome variables.

**Table 5:** Indirect effect of the structural model.

**Paths**	**Std. β**	**Std. error**	***t*** **-value**	**Decision**
SSS → SE → LS	0.119	0.020	5.984	Support
SSS → SGS → LS	0.057	0.017	3.363	Support
IR → SE → LS	0.068	0.017	4.021	Support
IR → SGS → LS	0.089	0.019	4.703	Support
SSS → SE → SGS	0.097	0.019	5.080	Support
IR → SE → SGS	0.056	0.014	4.085	Support

## Discussion and Conclusions

### Discussion

This research combines the SCCT and SOR models to construct a conceptual model that includes psychosocial cognition and the mental operation process and explores how to enhance the learning satisfaction of students from a process point of view. In the SCCT model, although the interactions among the individuals, their environments, and their behaviors are emphasized, there is a foreseeable gap in the formation of the internal psychological cognition of the individual and its reflection in the subsequent behavior and attitude under the influence of external stimuli. The addition of the SOR model can help us more rigorously explain the development process of the inner psychological cognition of students in a learning environment that receives external stimuli and its enhancement effect on learning satisfaction. The research results point out that the model has a good fit and a positive and significant effect on all paths, which further strengthens the rationality of the model in this research.

The research results point out that the institutional-level antecedent of a social support system has a positive and significant effect on self-efficacy and student generic skills. The findings of this research show that, if the university provides a more diverse or rich social support system, students will feel that they are valued by the school and obtain corresponding information and resources in the process of completing the course tasks and have the confidence and ability to do so. It has been found that the positive effect of social support on self-efficacy conforms to the research results from Liu et al. (2020) and Xu et al. (2021), which provides a second verification that, under the research background of the Asian area, social support can effectively improve the self-efficacy of students and enhance the generalization of the SCCT research and theory. Students are available to deal with various challenges faced with confidence and abilities, as well as obtaining a lot of learning experience from them. This is similar to the results of Malecki and Demaray (2003), Wang et al. (2015), and Orkibi et al. (2018), who stated that students who do not have self-directed learning skills in the process of achieving course tasks will not know how to do the same in the learning process, thus having more feelings of disability and helplessness (Yilmaz, 2017). The results of this study, similar to the results of the studies in the literature, indicate that a social support system is an important predictor of self-efficacy and student generic skills in the SCCT model.

Similarly, the research results point out that the individual-level antecedent of an interaction relationship has a positive and significant effect on self-efficacy and student generic skills as stated in hypothetical inference. Research findings provide clear information expressing that students, who continue to maintain and establish interaction relationships, can strengthen learning collaboration between peers through close social relationships, and acquire rich experience and skills in the learning process. The research results echo the research of Pike et al. (2012) and Peng (2019), emphasizing that the interpersonal and interaction relationships of students play an important role in campus life and enrich the generality of the application of social capital in the SCCT and SOR models. Despite the study from Pike et al. (2012) stating that only the influence of an interaction relationship on student learning outcomes was verified and no theoretical framework was added for discussion, the operational definition from Pike et al. (2012) was used as the antecedent in the theoretical framework in this study; the interaction relationship was confirmed to be available for not only improving student learning outcomes but also having substantial positive effects on psychological factors.

As some cross-cultural research results show, different from Western students, the learning environment of students in Eastern societies or Asian regions emphasizes the importance of relationships. Thus, the positive learning thoughts, feelings, and behaviors of students will be affected by the mutual links in their social relations (Chang et al., 2011). The hypothesis points out that self-efficacy will positively affect student generic skills. The research results are similar to those from Satoshi et al. (2009), that is, high self-efficacy can make the acquisition of generic skills and professional competence more accessible to students in a more effective way. The research results support this argument, and the role of self-efficacy as a mediator in the SCCT model has also been verified. These results are similar to previous studies (Doménech-Betoret et al., 2017; Liu et al., 2020; Xu et al., 2021). They all believe that they have higher self-efficacy. Students can increase their learning input in the learning situation set by the teacher. When students detect the improvement of their own generic skills, the satisfaction students have with their psychological needs will be affected (Pan, 2014). Similarly, many researchers have designed a sound research framework from the SCCT model (Liu et al., 2020), deducing that various internal and external learning process variables will affect students in their formation of a high degree of self-efficacy. Through a social support system and interaction relationships, in addition to enhancing the self-efficacy of internal learning motivation, students can also indirectly strengthen their professional competence and soft skills.

Finally, the research results show that self-efficacy and generic skills have a positive impact on learning satisfaction. This result is consistent with the final attitude cognition and behavioral response in the SCCT and SOR models proposed by scholars. The research findings are also similar to Kong and Yan (2014)'s research results, pointing out that learning satisfaction is related to the academic development achievements of students. Under the premise of learning self-efficacy and enhancement of generic skills, students can feel a high degree of academic achievement on their own, thereby enhancing their learning satisfaction (Nandi et al., 2015). This discovery provides significant support for both the SCCT model and the SOR model. These results correspond with those of Wu et al. (2019), Cupani et al. (2010), Zhai et al. (2020), and Fu et al. (2021); on the basis of the SCCT and SOR models, they believe that learning environment differences between stimulus and learning influence the learning status and learning activities of students, causing knowledge and skills-gaining to differ. Our findings are largely consistent with those from these prior studies, supporting the availability of the SCCT model across a range of theoretical frameworks. It shows the importance of cognitive psychology in the processing of external stimuli, and also proposes a more complete theoretical model and contribution to the SCCT model.

### Educational Practices

Practically, the results of this study may provide useful guidance for higher education institutions, faculties, administrators, and teachers on student learning satisfaction development. First, the social support system of a school has a significant effect on the enhancement of the self-efficacy and generic skills of students. It means that students pay attention to the changes in the learning environment if the school attaches great importance to them during the learning process. The social support system can play an effective role when students feel learning powerlessness, learning frustration, and helplessness. For example, the school provides more meta-media learning equipment, after-school tutoring mechanism, teacher's learning care, etc. With these tangible equipment and software and intangible psychological support, students can reduce their learning difficulties, improve their input in learning, and enhance their motivation to complete learning tasks.

Second, the study found that the interactions and social capital of students also have a clear positive impact on self-efficacy and generic skills. Interaction relationships and social capital are external connections maintained and established by students themselves. When the relationship between external connections becomes closer and more numerous, more resources, information, and knowledge can be effectively obtained, which is conducive for the cultivation of psychological functions and abilities. However, not all students can take the initiative to establish and cultivate their interaction relationships, especially their relationships with teachers; in other words, teachers or schools must provide more opportunities for interaction between teachers and students, with teachers moving beyond a passive role. This research suggests that schools or teachers can provide after-school consultation activities. With these consultations, teachers can fully understand the problems or learning difficulties faced by students and provide effective help. Furthermore, teachers can also provide more teamwork in the course, as these activities provide opportunities for students to communicate with each other and collaborate to solve classroom tasks, thereby strengthening the interaction between the three parties.

Third, the study found that student self-efficacy and generic skills not only have a significant effect on learning satisfaction but also play an important intermediary role in the model. Most previous studies emphasized practical knowledge or hard skills. However, students can clearly express the acquired explicit skills, but seldom mention them with higher implicit skills or emotional cognition. Thus, this study presented actual evidence pointing out that implicit skills or cognition are more helpful to improve the learning satisfaction of students. Therefore, this research suggests that schools should offer more general education courses related to majors and encourage teachers to carry out more functional teaching activities, which will help students develop more generic skills and enhance their satisfaction with learning.

### Limitations

The research results contribute to the literature on the SCCT and SOR models and student learning satisfaction; nevertheless, some limitations still exist and represent further research directions. First, the SSCT and SOR models have obtained considerable status in the psychological field, but only a few studies have considered the relationship between the building mechanism and learning satisfaction of undergraduate students in higher education. Although the building mechanism (social support system and interaction relationship) was constructed with reference to the SCCT and SOR models in this study and important learning theories can be derived from the research results, other motivation theories, such as attribution theory, self-efficacy theory, and hierarchy needs theory must still be applied to explain how to trigger learning in undergraduate students. Thus, it is suggested that future research can utilize different theoretical models in order to identify relevant psychological dimensions influencing the learning satisfaction of students. Second, this study required students to self-report details on their psychological building mechanism as the indicator, mainly because actual data is confidential and not easily obtained. However, errors may exist in the self-statements students made of their psychological status. The link between the building mechanism and learning satisfaction may be better understood if the actual psychological status of students could be assessed, with due consideration for research ethics. Besides, this study suggests that future researchers should include interview contents and observations by students on the learning status in their studies to support the research results and make a comprehensive judgment. Third, due to restrictions of time and space, only 14 universities were sampled in this study, with 800 valid questionnaires in total. Future research could explore and compare other groups, in addition to expanding the quantity of samples and improving the research representativeness, so as to provide additional insights relevant to higher education policy. Finally, Wong (2020) put forward that there may be differences in after-school and in-class psychological cognitive results produced by students, and there is an unsolved black box between them. However, this classification was not analyzed in this study. Thus, in this study, the researchers also suggest that future studies compare the after-class and in-class differences and offer more valuable insights into the unsolved black box.

## Data Availability Statement

The raw data supporting the conclusions of this article will be made available by the authors, without undue reservation.

## Ethics Statement

The studies involving human participants were reviewed and approved by University of Taipei. The patients/participants provided their written informed consent to participate in this study.

## Author Contributions

This study is a joint work of the four authors. MP and GZ contributed to the ideas of educational research, collection of data, and empirical analysis. MP, GZ, and XY contributed to the data analysis, design of research methods, and tables. XY and YY participated in developing a research design, writing, and interpreting the analysis. All four authors contributed to the literature review and conclusions.

## Conflict of Interest

The authors declare that the research was conducted in the absence of any commercial or financial relationships that could be construed as a potential conflict of interest.

## Publisher's Note

All claims expressed in this article are solely those of the authors and do not necessarily represent those of their affiliated organizations, or those of the publisher, the editors and the reviewers. Any product that may be evaluated in this article, or claim that may be made by its manufacturer, is not guaranteed or endorsed by the publisher.
